# tRNA^Ini^_CAT_ inhibits proliferation and promotes apoptosis of laryngeal squamous cell carcinoma cells

**DOI:** 10.1002/jcla.23821

**Published:** 2021-05-28

**Authors:** Jun Li, Zhisen Shen, Lin Luo, Dong Ye, Hongxia Deng, Shanshan Gu, Chongchang Zhou

**Affiliations:** ^1^ Department of Otorhinolaryngology, Head and Neck Surgery The Affiliated LiHuili Hospital Ningbo University Ningbo China; ^2^ Department of Biochemistry and Molecular Biology Zhejiang Key Laboratory of Pathophysiology Ningbo University School of Medical Ningbo China

**Keywords:** biomarkers, laryngeal squamous cell carcinoma, tRNA, tRNA expression profile, tRNA^Ini^_CAT_

## Abstract

**Background:**

Laryngeal squamous cell carcinoma (LSCC) brings a heavy blow to the patient's voice. Transfer RNA (tRNA) is a common RNA, the roles of tRNAs in LSCC are largely unknown.

**Methods:**

The tRNA expression profile in LSCC tissues and adjacent normal tissues was measured by a tRNA qRT‐PCR array. The expression level of tRNA^Ini^_CAT_ in LSCC tissues and plasmas was detected by qRT‐PCR. The receiver operating characteristic (ROC) curve was established. tRNA^Ini^_CAT_ was upregulated by a lentivirus vector in the LSCC cell line. Moreover, tRNA^Ini^_CAT_ was upregulated in LSCC xenograft nude mouse model and the xenografts were used for pathological analysis and transmission electron microscope (TEM) observation.

**Results:**

The top 10 upregulated tRNAs were tRNA^Lys^_CTT_‐1, tRNA^Leu^_TAA_, tRNA^Phe^_GAA_, tRNA^Leu^_CAG_, tRNA^Tyr^_ATA_, tRNA^Met^_CAT_, tRNA^Tyr^_GTA_‐1, tRNA^Thr^_CGT_, tRNA^Tyr^_GTA_‐2, tRNA^Ala^_AGC_; and the top 10 downregulated tRNAs were tRNA^Ini^_CAT_, mt‐tRNA^Glu^_TTC_, tRNA^Val^_CAC_‐3, mt‐tRNA^Trp^_TCA_, mt‐tRNA^Tyr^_GTA_, mt‐tRNA^Lys^_TTT_, mt‐tRNA^Thr^_TGT_, mt‐tRNA^Asp^_GTC_, mt‐tRNA^Asn^_GTT_, mt‐tRNA^Pro^_TGG_. tRNA^Ini^_CAT_ was downregulated in LSCC tissues and plasma. The area under the ROC curve (AUC) in LSCC tissues and the plasma of patients with LSCC was 0.717 and 0.808, respectively. tRNA^Ini^_CAT_ inhibited LSCC cell proliferation and promoted apoptosis. The *in vivo* results showed that tRNA^Ini^_CAT_ inhibited the growth of the xenografts and promoted apoptosis.

**Conclusions:**

This is the first study to provide tRNA expression profiles for LSCC tissues. tRNA^Ini^_CAT_ may be used as a new biomarker for the early diagnosis of LSCC. tRNA^Ini^_CAT_ inhibits cell proliferation and promotes apoptosis *in vitro* and *in vivo*.

## Graphical abstract

tRNAIniCAT inhibited the proliferation and induced the apoptosis of LSCC cells in vivo. A: Nude mouse model of xenograft. (a) NC. (b) Low dose. (c) High dose. B: Tumor weight in high‐dose tRNAIniCAT‐treated groups were significantly lower than the NC group. *p<0.05. C: There was no significant difference in tumor volume between each group. D: There was no significant difference in the weight of mice between each group. E: Pathological examination showed that tumor necrosis in the high‐dose treatment group was markedly increased compared with that observed in the NC group (magnification ×100). (a) NC. (b) Low dose. (c) High dose. F: TEM results showing that autophagy and apoptosis were more observed in the treatment group (b and c) (magnification ×20,000). (a) NC. (b) Low dose. (c) High dose.

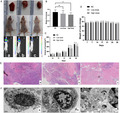

## 1 INTRODUCTION

Laryngeal cancer is a common malignant tumor of the head and neck. It is common among men aged 50–70 years. Its incidence is 1.5–3/100,000, accounting for approximately 1% of all malignant tumors. Laryngeal squamous cell carcinoma (LSCC) is the most common type, accounting for approximately 90% of cases.^1^ At present, the incidence of laryngeal cancer is increasing. Treatment is mainly through surgical resection, combined with preoperative or postoperative chemotherapy, radiotherapy, and other comprehensive therapies. However, vocal disorders occur following surgery, affecting the quality of life of patients. Globally, more than 80,000 patients with laryngeal cancer expire each year.^2^ Because the symptoms of early laryngeal cancer are not obvious, early detection is very important to reduce the hazard of laryngeal cancer. On the one hand, it can improve the survival rate of patients after surgery, on the other hand, it preserves the voice function of the larynx as much as possible to reduce postoperative complications.

Transfer RNA (tRNA) is a universal RNA present in all forms of life. A mature tRNA is characterized by its secondary structure which consists of three loops and four stems: D‐loop, anticodon loop, T‐loop, and D‐stem, anticodon stem, T‐stem, acceptor stem; as well as an L tertiary structure maintained by hydrogen bonds.^3^ As a basic component of the translation process, tRNA transport amino acids to the ribosome; the genetic information of the nucleotide sequence is transformed into the corresponding polypeptide chain in the manner of codon (mRNA)‐anticodon (tRNA) interaction^4^; this process is essential to maintain the normal life activities of the body. More than half of RNA modifications occur in tRNA.^5^ These modifications affect the structure, stability, and functionality of tRNA, leading to widespread cellular effects; of those, methylation modification is the most common effect.^6^

Early studies reported that tRNA plays only the role of a "porter", and does not possess regulatory function. Nevertheless, as research progresses, tRNA have been found to be involved in various physiological and pathological processes, including cancer, diabetes, and neuronal disease.^7^, ^8^, ^9^ The regulatory role of tRNA in LSCC remains unknown and there is no complete tRNA expression spectrum on this disease. This is the very first study to provide tRNA expression profiles for LSCC tissues. We analyzed the diagnostic value of tRNA^Ini^_CAT_ through detecting the expression level in tissues and plasma. In addition, we proved that tRNA^Ini^_CAT_ acts as a tumor inhibitor in LSCC through cell culture and nude mouse xenograft experiments.

## 2 MATERIALS AND METHODS

### 2.1 Collection of specimens

Patients with LSCC who underwent otorhinolaryngology, head and neck surgery at LiHuili Hospital affiliated to Ningbo University (Ningbo, China) from January 2013 to April 2019 without receiving radiotherapy, chemotherapy, or other antitumor treatments were selected. Tumor tissue and adjacent normal tissue samples were obtained from surgery. All LSCC samples were confirmed by at least two senior pathology experts. Healthy peoples' plasma and plasma from patients with LSCC were also obtained on the day before surgery and 7 days after surgery. Tumor staging was determined according to the American Joint Committee on Cancer tumor‐lymph node‐metastasis (TNM) staging (2017, 8th edition). This study was approved by the Ethics Committee of Human Research of Ningbo University (No.KY2015PJ012). Written informed consent was provided by all subjects.

### 2.2 Total RNA preparation and synthesis of cDNA

The TRIzol reagent (Invitrogen, Carlsbad, CA, USA) and TRIzol LS reagents (Invitrogen) was used to extract the total RNA of tissues/cells and plasma through the SmartSpec Plus spectrophotometer (BioRad, Hercules, CA, USA). The purity and concentration of total RNA were determined. The RNA quality control standards were as follows: A260:A230 ratio >1.7; A260:A280 ratio 1.8–2.1. According to the rtStar™ tRNA‐optimized First‐Strand cDNA Synthesis Kit (Arraystar, Rockville, MD, USA) instructions, 2 μg total RNA was used for demethylation reactions and cDNA synthesis. For assessing the quality of synthetic cDNA, 1 μl RNA Spike‐in quantitative polymerase chain reaction (qPCR) Primer Mix, 2 μl cDNA, 5 μl GoTaq qPCR Mixture (Promega, Madison, WI, USA) and 2 μl enzyme‐free water were added; only samples with quantification cycle (*C*_q_) values <30 were used for qPCR detection.

### 2.3 qPCR

The cDNA was diluted with enzyme‐free water (1:20). The Applied Biosystems 7900 real‐time PCR system (Thermo‐Fisher, USA) was used to qPCR. The primers of 88 tRNAs were bought from Arraystar (Rockville, MD, USA). U6 was used as a control, and the relative expression level of tRNA was determined by the ΔΔ*C*_q_ method. U6 primers were purchased from BGI (Shanghai, China). The U6 sequences were as follows: justice, 5′‐ GCTTCGGCAGCACATATACTAAAAT‐3′; Antimony 5′‐ CGCTTCACGAATTTGCGTCAT‐3′.

### 2.4 Cell culture and transfection

The LSCC cell line AMC‐HN‐8 was purchased from BeNa Culture Collection (Shanghai, China). The cells were cultured in RPMI 1640 (HyClone, Logan, UT, USA) with 10% fetal bovine serum (PAN Biotech, Aidenbach, Germany) at 37°C and 5% CO_2_. Cells were counted using a TC10 automatic cell counter (BioRad). The lentivirus vector was purchased from GenePharma (Shanghai, China). Stably transfected cells were selected through puromycin. The tRNA^Ini^_CAT_ overexpression lentivirus vector is termed LV3‐tRNA^Ini^_CAT_, Sequences: 5′‐ AGCAGAGTGGCG CAGCGGAAGCGTGCTGGGCCCATAACCCAGAGGTCGATGGATCGAAACCATCCTCTGCTA‐3′, LV3‐NC used as a control, sequence: 5′‐ TTCTCCGAACGTGTCACGT‐3′.

### 2.5 ^3^H‐TdR penetration test

AMC‐HN‐8 cells were digested with trypsin to form a single‐cell suspension and inoculated on a six‐well plate. A culture medium containing ^3^H‐TdR was added for 16 h, and the cells were digested and collected. Finally, a Micro Beta 2450 liquid scintillation counter (Perkin Elmer, Waltham, Mass, USA) was used to determine the counts per minute (cpm), and the smaller the cpm value, the greater the inhibition of cell proliferation.

### 2.6 Cell cycle and apoptosis analysis

The cells were serum‐starved to synchronize the cell cycle. Subsequently, the cells were collected, washed with phosphate‐buffered saline (PBS), and fixed in 70% ethanol at −20°C for 24 h. Next, staining with DNA dye solution was performed using the Cell Cycle Detection Kit (KeyGEN, Jiangsu, China), and the cells were incubated for 30 min. Lastly, BD FACSCanto II flow cytometry (BD Biosciences, San Jose, CA, USA) Testing was performed. Cells were digested with trypsin (no ethylenediaminetetraacetic acid) into a single‐cell suspension and resuspended in binding buffer. The cells were then stored at room temperature. Using an Annexin V‐FITC/PI apoptosis kit (CoWin Biosciences, Beijing, China), the cells were stained for 15 min without light, and the rate of apoptosis was detected using BD FACSCanto II flow cytometry (BD Biosciences).

### 2.7 Nude mouse model of xenograft

All animal studies were conducted in accordance with the animal protocol approved by the Institutional Animal Care and Use Committee of Zhejiang Chinese Medical University (Hangzhou, Zhejiang, China). BALB/c female nude mice were purchased from Charles River (Beijing, China) and raised in the experimental animal center of Zhejiang Chinese Medical University. The mice were anesthetized with pentobarbital sodium (45 mg/kg) and inoculated with AMC‐HN‐8 cell suspension subcutaneously on the front of the neck. The mice were randomly divided into three groups (9 or 10 mice per group). Xenograft growth was observed using an IVIS Lumina XRMS *in vivo* imaging system (Perkin Elmer) after 1 week. The mice in each group were injected with 50 μl of corresponding solution within the xenograft (twice per week for 21 consecutive days). The weight of mice and volume of xenograft were measured twice per week. The day after drug withdrawal, the mice were sacrificed and the xenograft was resected and weighed.

### 2.8 Hematoxylin‐eosin (HE) staining and transmission electron microscopy (TEM)

The xenograft tissue was fixed in 10% formalin, embedded in paraffin, cut into sections (thickness: 8 µm) and baked at 45°C for 5 h. Subsequently, the tissues were dewaxed with xylene for 30 min, treated with different concentrations of ethanol (100%, 90%, 70%); hydrated with distilled water, stained with hematoxylin for 15 min, differentiated in ethanol and ammonia, dehydrated with ethanol, stained with eosin, dehydrated again with ethanol, and washed with xylene. Tissue morphology was observed by optical microscopy.

The xenograft tissue was fixed with 2.5% glutaraldehyde for 1 h and washed with phosphate‐buffered saline for 1 h. Then was subsequently fixed in 1% osmium acid for 30 min–1 h, and dehydrated in ethanol and acetone. Finally, the tissue was embedded in epoxy resin and stained with uranyl acetate and lead citrate. The Hitachi H‐7650 TEM (Tokyo, Japan) was used to observe the changes in autophagy and apoptosis morphology.

### 2.9 Statistical analysis

The SPSS version 20.0 software (IBM Corp., Armonk, NY, USA) and GraphPad 8.0 (San Diego, CA, USA) were used for data analysis. The relative expression level of tRNA^Ini^_CAT_ between LSCC tissues and adjacent normal tissues was analyzed by paired sample t test. One‐way analysis of variance was used to analyze the level of tRNA^Ini^_CAT_ expression in the preoperative plasma, postoperative plasma of patients with LSCC and plasma from healthy volunteers. The diagnostic value was assessed using the receiver operating characteristic (ROC) curve. *p* < 0.05 denoted statistically significant differences.

## 3 RESULTS

### 3.1 Differences in tRNA expression profile of LSCC tissues

**FIGURE 1:**
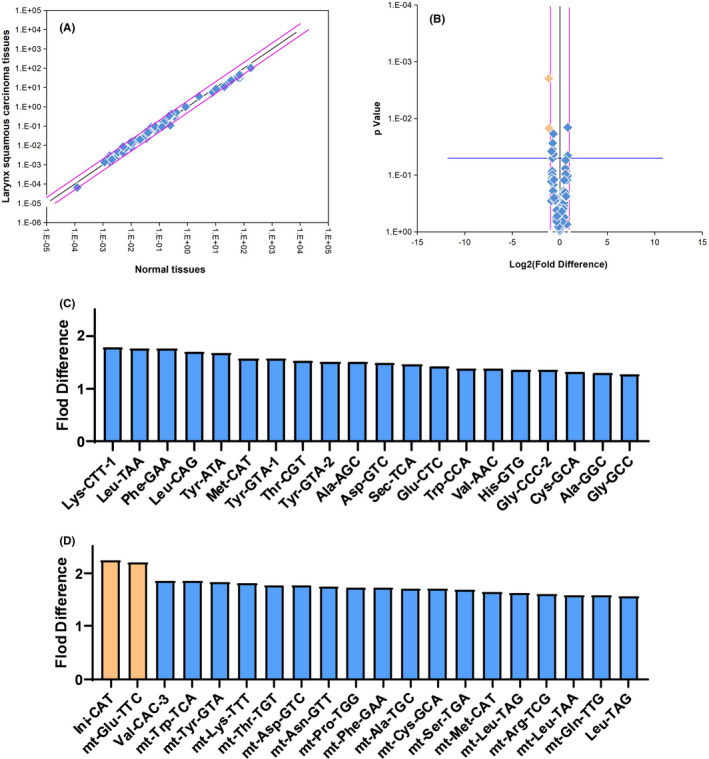
tRNA expression profile in LSCC tissues and adjacent normal tissues. A, Scatter diagram. The black line indicates fold changes of 1 (no difference). The pink lines indicate the fold‐change threshold (2.0). B, Volcano plots. The black line indicates a fold‐change value of 1. The pink lines indicate the threshold of the fold‐change (2.0). The blue line indicates the p value cutoff (0.05). The orange squares correspond to two tRNAs with significant differences in Figure 1D. C, upregulated tRNAs in LSCC tissues with highest fold difference (Top 20). D, downregulated tRNAs in LSCC tissues with highest fold difference (Top 20). tRNAs with a fold difference more than 2 were marked orange

The expression levels of 88 tRNAs (including 66 cytoplasmic tRNAs and 22 mitochondrial tRNAs) were detected in 8 pairs of LSCC tissues and adjacent normal tissues. The results showed that the expression profiles of tRNA in LSCC tissues were different from those observed in adjacent normal tissues (Figure 1A,B). We found that two tRNAs were significantly differentially expressed (Folding multiple >2) in LSCC tissues versus adjacent normal tissues: tRNA^Ini^_CAT_ (fold = −2.24) and mt‐tRNA^Glu^_TTC_ (fold = −2.21), which were marked orange (Figure 1B). In LSCC tissues, the top 10 upregulated tRNAs were tRNA^Lys^_CTT_‐1, tRNA^Leu^_TAA_, tRNA^Phe^_GAA_, tRNA^Leu^_CAG_, tRNA^Tyr^_ATA_, tRNA^Met^_CAT_, tRNA^Tyr^_GTA_‐1, tRNA^Thr^_CGT_, tRNA^Tyr^_GTA_‐2, tRNA^Ala^_AGC_ (Figure 1C); and the top 10 downregulated tRNAs were tRNA^Ini^_CAT_, mt‐tRNA^Glu^_TTC_, tRNA^Val^_CAC_‐3, mt‐tRNA^Trp^_TCA_, mt‐tRNA^Tyr^_GTA_, mt‐tRNA^Lys^_TTT_, mt‐tRNA^Thr^_TGT_, mt‐tRNA^Asp^_GTC_, mt‐tRNA^Asn^_GTT_, mt‐tRNA^Pro^_TGG_ (Figure 1D).

### 3.2 Low expression of tRNA^Ini^_CAT_ was detected in LSCC tissues and plasma and potential diagnostic values

**FIGURE 2:**
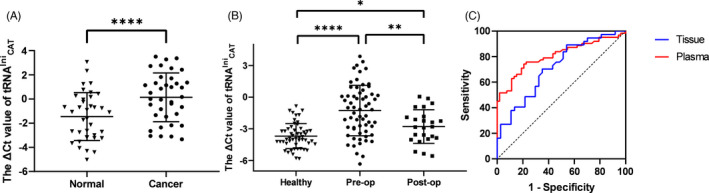
tRNAIniCAT expression levels and ROC curve of LSCC tissues and plasma samples. A, The expression levels of tRNAIniCAT in LSCC were significantly lower than those in the adjacent normal tissues (*n* = 37; ****p < 0.0001). B, The expression levels of tRNAIniCAT in the preoperative plasma (*n* = 62) were significantly lower than those noted in postoperative plasma (*n* = 24) and healthy human plasma (*n* = 53). Meanwhile, the expression levels in postoperative plasma were significantly lower than those observed in healthy human plasma. *p < 0.05; **p < 0.01; ****p < 0.0001. C, ROC curve of tRNAIniCAT in tissues and plasmas

To validate the results of the expression profile, tumor and adjacent normal tissue samples were collected from a total of 37 patients with LSCC through qRT‐PCR to determine the relative expression levels of tRNA^Ini^_CAT_. As shown in Figure 2A, the expression levels of tRNA^Ini^_CAT_ in tumor tissues were significantly lower than those measured in adjacent normal tissues (*p* < 0.0001). By exploring the relative expression levels of tRNA^Ini^_CAT_ in plasma, we obtained consistent results with those reported for tumor tissues. The expression levels of tRNA^Ini^_CAT_ in the preoperative plasma of patients with LSCC were significantly lower than those noted in postoperative plasma (*p* = 0.0032) and healthy human plasma (*p* < 0.0001). Meanwhile, the expression levels in postoperative plasma were significantly lower than those observed in healthy human plasma (*p* = 0.0458) (Figure 2B). The area under the ROC curve (AUC) of tRNA^Ini^_CAT_ was 0.808 in plasmas, with 74.2% sensitivity and 79.3% specificity, the cutoff was −2.9. The AUC in tissues was 0.717, with 89.2% sensitivity and 46% specificity, the cutoff was 0.8 (Figure 2C).

### 3.3 Upregulation of tRNA^Ini^_CAT_ inhibits proliferation and promotes apoptosis of LSCC cell

**FIGURE 3:**
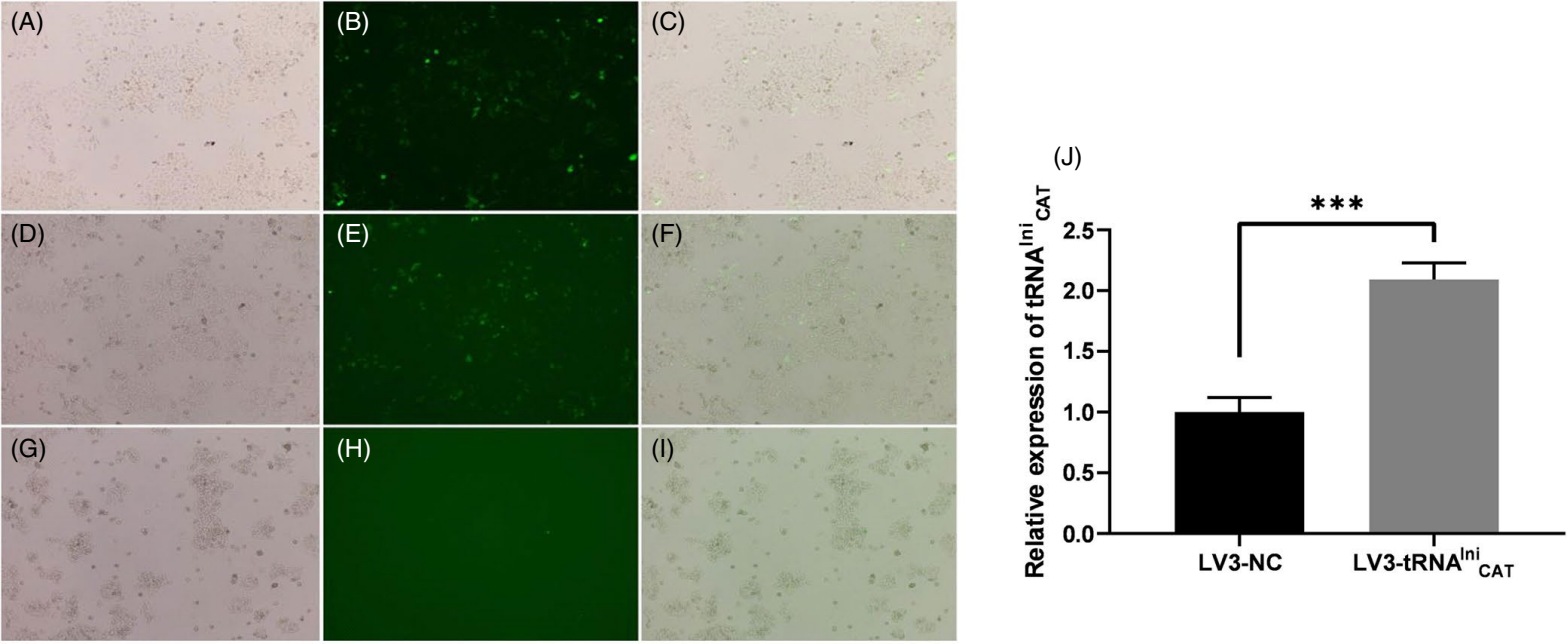
Fluorescence microscopy findings showing GFP fluorescence expression in AMC‐HN‐8 cells after transfection with LV3‐tRNAIniCAT and LV3‐NC (magnification: ×100). A–C, LV3‐tRNAIniCAT groups; D–F, LV3‐NC groups; G–I, blank. J, Overexpression of tRNAIniCAT in AMC‐HN‐8 cell lines was analyzed by qRT‐PCR (*n* = 3, ***p < 0.001)

**FIGURE 4:**
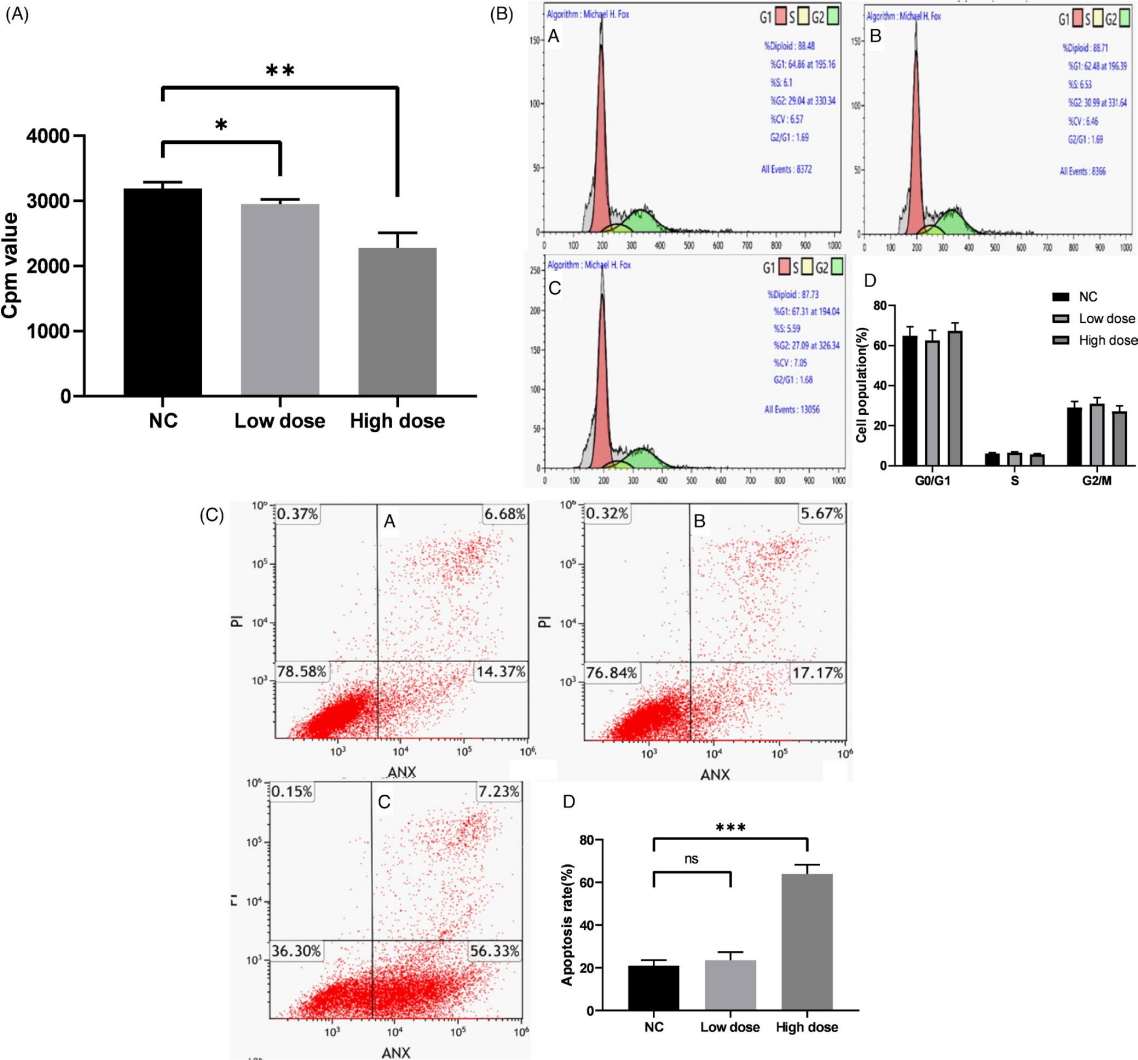
tRNAIniCAT inhibits proliferation and promotes apoptosis of AMC‐HN‐8 cells. A, A liquid scintillation counter was used to measure the counts per minute (cpm) of the three groups (*n* = 3, *p < 0.05; **p < 0.01). B, The overexpression of tRNAIniCAT did not have a significant effect on the AMC‐HN‐8 cell cycle (*n* = 3). (a) NC. (b) Low dose. (c) High dose. (d) Histogram. C, The effect of high overexpression of tRNAIniCAT on the apoptosis of AMC‐HN‐8 cells (n=3, ***p < 0.001, ns, no significance). (a) NC. (b) Low dose. (c) High dose. (d) Histogram

AMC‐HN‐8 cells were transfected by LV3‐tRNA^Ini^_CAT_ to upregulate tRNA^Ini^_CAT_ to assess the role of tRNA^Ini^_CAT_ in tumor progression. Following transfection of LV3‐tRNA^Ini^_CAT_ and LV3‐NC in AMC‐HN‐8 cells, green fluorescent protein fluorescence expression was observed (Figure 3A–I). The expression levels of tRNA^Ini^_CAT_ in the cells showed a 2.1‐fold increase (Figure 3J). The ^3^H‐TdR penetration test results showed that overexpression of tRNA^Ini^_CAT_ significantly inhibited cell proliferation compared with the NC group (Figure 4A). For further exploration of the potential regulatory mechanisms of tRNA^Ini^_CAT_, cell cycle and apoptosis were assessed by flow cytometry. The upregulated tRNA^Ini^_CAT_ did not have a significant effect on the cell cycle (Figure 4B). Furthermore, the results of the apoptosis analysis showed that transfection of LV3‐tRNA^Ini^_CAT_ significantly promoted apoptosis in AMC‐HN‐8 cells (Figure 4C). Collectively, these results indicated that tRNA^Ini^_CAT_ inhibited proliferation and promoted apoptosis of LSCC cell.

### 3.4 tRNA^Ini^_CAT_ inhibits growth of LSCC xenograft

**FIGURE 5:**
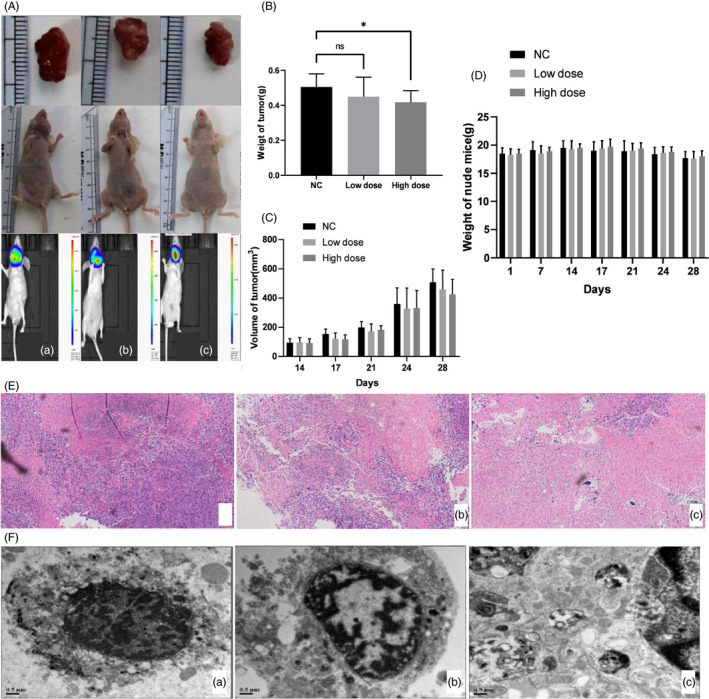
tRNA^Ini^_CAT_ inhibited the proliferation and induced the apoptosis of LSCC cells in vivo. A, Nude mouse model of xenograft. (a) NC. (b) Low dose. (c) High dose. B, Tumor weight in high‐dose tRNA^Ini^_CAT_‐treated groups was significantly lower than the NC group. *p < 0.05. C, There was no significant difference in tumor volume between each group. D, There was no significant difference in the weight of mice between each group. E, Pathological examination showed that tumor necrosis in the high‐dose treatment group was markedly increased compared with that observed in the NC group (magnification ×100). (a) NC. (b) Low dose. (c) High dose. F, TEM results showing that autophagy and apoptosis were more observed in the treatment group (b and c) (magnification ×20,000). (a) NC. (b) Low dose. (c) High dose

We used a LSCC xenograft mouse model to investigate the antitumor role of tRNA^Ini^_CAT_ *in vivo*. The mice were divided into three groups: LV3‐tRNA^Ini^_CAT_‐high dose, LV3‐tRNA^Ini^_CAT_‐low dose and LV3‐NC (Figure 5A). The results indicated that upregulation of tRNA^Ini^_CAT_ significantly inhibited xenograft growth (Figure 5B). Among them, the weight of the xenograft in the high‐dose group was significantly smaller than that in the NC group. During the experiment, there was no significant difference in xenograft volume (Figure 5C) and mouse weight (Figure 5D) in these groups. Pathological findings indicated that the treatment of LV3‐tRNA^Ini^_CAT_ significantly increased tissue necrosis in xenograft, showing a certain role of tRNA^Ini^_CAT_ in promoting apoptosis (Figure 5E). Through TEM, apoptotic cells were shown to have special structural characteristics. The autophagy and apoptosis of xenograft in the LV3‐tRNA^Ini^_CAT_ treatment group were significantly increased, the phagocytic structure in the bubble was degraded, and the nucleus had shrunk significantly (Figure 5F). Therefore, we concluded that tRNA^Ini^_CAT_ promotes LSCC cell apoptosis *in vivo*.

## 4 DISCUSSION

In the past, the ncRNA expression spectrum of LSCC mainly focused on miRNA and lncRNA, our previous research revealed that miR‐34a,^10^ AC026166.2‐001^11^ and RP11‐169D4.1‐001^12^ were closely related to the occurrence of LSCC. A recent study found that lncRNA MALAT1 was overexpressed in LSCC tissues and highly correlated with the 5‐year survival of patients; it triggers the resistance of LSCC to chemotherapy drugs by promoting metastasis and inhibiting tumor cell apoptosis.^13^ However, there has not been any report about tRNA in LSCC. tRNA was widely regarded as a housekeeping gene with limited regulatory function. Compared with other additional functions of tRNA,^14^ the researchers understand that tRNA acts an adaptor for protein synthesis better.

In recent years, a growing body of evidence suggests that tRNA and its derivatives are dysregulated and participate in the pathogenic process of cancer.^15^ Mutations in tRNA and the involvement of co‐proteins produced by tRNA biogenesis and modification were found to be associated with cancer.^16^, ^17^, ^18^ Meanwhile, mutations in mitochondrial tRNA cause mitochondrial dysfunction which is also associated with tumorigenesis.^19^ Studies have found that tRNA synthesis was controlled by various oncogenic and tumor suppressor genes. Ras^20^ and c‐Myc^21^ facilitate RNA Pol III transcription; however, Rb^22^ and p53^23^ inhibit RNA Pol III transcription, leading to severe dysregulation of tRNA levels in multiple types of cancer. In addition, in several types of cancer, tRNA modified enzymes can add some tRNA modification, which changes their codon preferences and leads to an increase in protein expression levels of mRNA with "preference" codons.^24^, ^25^

Numerous studies have confirmed the important role of tRNA in regulating gene expression. Change in bacterial activity tRNA content plays an adaptive role with environmental signal changes.^26^ The binding of tRNA to cytochrome C inhibits the effects of cytochrome C and apoptotic proteases, thereby inhibiting apoptosis and enzyme activity.^27^ Overexpression of the initial tRNA (tRNA_i_^Met^) was observed in normal mammary epithelial cells. Changes in the whole cell tRNA expression levels accelerate the speed of cell proliferation.^28^ In carcinoma‐associated fibroblasts, tRNA_i_^Met^ promotes tumor proliferation and angiogenesis.^29^ Studies revealed that tRNA^Ile^ expression was higher in breast cancer‐associated fibroblasts than in normal fibroblasts.^30^ These abnormally expressed tRNA molecules are expected to be new prognostic markers for such diseases.^31^ tRNA^Glu^_UUC_ and tRNA^Arg^_CCG_ are highly expressed in breast cancer and enhance tumor invasion. EXOSC2 and GRIPAP1 are downstream target proteins of tRNA^Glu^_UUC_ to promote the progression of cancer invasion and metastasis.^32^ In human epidermal growth factor receptor 2‐positive breast cancer cell lines, free tRNA^Leu^ may interact with human epidermal growth factor receptor 3 (ErbB3) and binding protein (EBP1), which in turn activates the ErbB2/ErbB3 signaling pathways, and ultimately promotes cancer cell proliferation.^33^

In the current study using a tRNA qRT‐PCR array, the expression profiles of tRNA in LSCC and normal tissues were found to differ. Consistent with the profiling result, the present study confirmed the lower expression levels of tRNA^Ini^_CAT_ in LSCC tissue and plasma samples (Figure 2A,B). More importantly, we first identified its potential diagnostic value. As a biomarker, the AUC of tRNA^Ini^_CAT_ in plasma is 0.808, the sensitivity and specificity were 74.2% and 79.3%, respectively (Figure 2C). Then we explored its impact on LSCC cells *in vitro* and *in vivo*. Firstly, we used a LV3‐tRNA^Ini^_CAT_ lentivirus to upregulate tRNA^Ini^_CAT_ in LSCC cells (Figure 3). The ^3^H‐TdR penetration test results showed that overexpression of tRNA^Ini^_CAT_ inhibited cell proliferation (Figure 4A). Moreover, the results of flow cytometry showed that the upregulation of tRNA^Ini^_CAT_ did not affect the cell cycle (Figure 4B), but boosted the cell apoptosis (Figure 4C). Lastly, we reached the same conclusion based on experiments in BALB/c mouse xenografts, tRNA^Ini^_CAT_ inhibited tumor growth and promoted tumor cell apoptosis *in vivo*. Overexpression of tRNA^Ini^_CAT_ significantly decreased the weight of the xenografts (Figure 5B). By pathological examination and TEM, we found that overexpression of tRNA^Ini^_CAT_ significantly increased tumor cell apoptosis and autophagy (Figure 5E,F).

Our research suggested that tRNA, like other ncRNA, may have regulatory functions in the process of tumor gene expression. Due to the key role of tRNA in the process of protein translation, its imbalance may lead to the imbalance of some signal proteins. The differences in tRNA expression profiles of LSCC tissues indicate that tumor cells may regulate the expression of some promoters in tumor progression by regulating tRNA levels. The results of TEM showed that the apoptosis of xenograft cells increased significantly which implied that tRNA^Ini^_CAT_ may affected the expression of signaling proteins in apoptosis‐related pathways. However, this needs further research to verification.

In conclusion, our study provided a comprehensive tRNA expression spectrum for LSCC. We found that tRNA^Ini^_CAT_ may be used as a new biomarker for LSCC. tRNA^Ini^_CAT_ acted as a tumor suppressor in LSCC.

## CONFLICT OF INTEREST

The authors declare no conflict of interest.

## Data Availability

All data are included in this article.
